# 280. A cross-sectional pilot study on the relationship of gut microbiota pattern to immunogenicity of influenza vaccine in lupus patients

**DOI:** 10.1093/ofid/ofad500.352

**Published:** 2023-11-27

**Authors:** Nathathai Kiewsanuan, Pintip Ngamjanyaporn, Prapaporn Pisitkun, Sunchai Payungporn, Vorthon Sawaswong, Prangwalai Chanchaem, Arnatchai Maiuthed, Porpon Rotjanapan

**Affiliations:** Faculty of Medicine Ramathibodi Hospital, Mahidol University, Bangkok, Krung Thep, Thailand; Faculty of Medicine Ramathibodi Hospital, Mahidol University, Bangkok, Krung Thep, Thailand; Faculty of Medicine Ramathibodi Hospital, Mahidol University, Bangkok, Krung Thep, Thailand; Faculty of Medicine Chulalongkorn University, Bangkok, Krung Thep, Thailand; Faculty of Medicine Chulalongkorn University, Bangkok, Krung Thep, Thailand; Faculty of Medicine Chulalongkorn University, Bangkok, Krung Thep, Thailand; Faculty of Pharmacy, Mahidol University, Bangkok, Krung Thep, Thailand; Faculty of Medicine Ramathibodi Hospital, Mahidol University, Bangkok, Krung Thep, Thailand

## Abstract

**Background:**

Immunosuppressive therapy, the nature of the autoimmune disease, and different gut microbiome patterns may affect influenza vaccination responses of lupus patients. Thus far, the gut microbiome patterns in this population in response to influenza vaccination have yet to be studied extensively. So, this study aims to evaluate the gut microbiome pattern associated with influenza vaccination response and assess the associated factors that may affect different gut microbiome patterns among lupus patients.

The bar plot shows relative abundances of gut microbiome at the phylum level in lupus patients and healthy controls.

**Figure:**
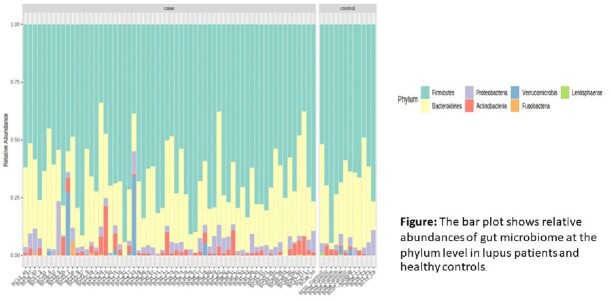

**Methods:**

The cross-sectional pilot study was conducted at Ramathibodi Hospital during 2021-2022. This study is a substudy of another cohort study investigating influenza vaccination response to different dosing regimens, randomly into the high-dose (HD) and standard-dose (SD) quadrivalent influenza vaccination. The patient’s demographics and other relevant information were retrieved. Fecal samples were collected at enrollment for microbiota study. Immunogenicity to vaccination was determined via seroprotection and seroconversion rates.

**Results:**

A total of 62 participants were enrolled. Most patients were female (93.55%). The median (IQR) ages among SD and HD groups were 35.5 (28-42.5) and 34 (25-43) (P=0.878) years old. 18 (29.03%) were taking high-level immunosuppressive treatment (HI) received SD, and 18 (29.03%) were taking low-level immunosuppressive treatment (LI) received SD. There was no significant difference in gut microbiota patterns among all patient groups. Firmicutes were the most abundant phylum, and Bacteroides were the most abundant genera in all patient groups. The alpha diversity was more prominent in the seroconversion groups but no statistical differences (P=0.260 in Shannon indexes). Multivariate logistic regression analysis revealed the LI group (AOR: 7.39, 95% CI 1.11 to 49.36; P=0.039), presence of Bacteroides (AOR 1.16, 95% CI 1.01 to 1.33; P=0.031) were associated with seroconversion of influenza vaccine.

**Conclusion:**

The gut microbiota patterns of lupus patients were not different from healthy controls nor vaccination response. Therefore, the immunosuppressive therapy did not affect the gut microbiota patterns in lupus patients, and an HD of influenza vaccine may not be necessary.

**Disclosures:**

**All Authors**: No reported disclosures

